# Elevated expression of eukaryotic translation initiation factor 3H is associated with proliferation, invasion and tumorigenicity in human hepatocellular carcinoma

**DOI:** 10.18632/oncotarget.10222

**Published:** 2016-06-22

**Authors:** Qian Zhu, Guo-Liang Qiao, Xiao-Chuan Zeng, Yun Li, Jian-Jun Yan, Rui Duan, Zhi-Yong Du

**Affiliations:** ^1^ Department of General Surgery, Jingmen First People's Hospital, Jingmen 448000, Hubei Province, China; ^2^ Department of Medical Oncology, Capital Medical University Cancer Center, Beijing Shijitan Hospital, Beijing, 100038, China; ^3^ First Department of Hepatic Surgery, Eastern Hepatobiliary Surgery Hospital, Second Military Medical University, Shanghai 200438, China; ^4^ Department of Hepatobiliary Surgery, Central Hospital of Wuhan, Wuhan 430014, Hubei Province, China

**Keywords:** hepatocellular carcinoma, initiation factors, microarray, prognosis, tumor progression

## Abstract

**Aim:**

We studied the role of eukaryotic translation initiation factor 3 subunit H (EIF3H) in hepatocellular carcinoma (HCC) progression.

**Results:**

High *EIF3H* expression was observed in 50.23% patients. Upregulation of *EIF3H* is an independent predictor for greater rates of cancer recurrence and shorter overall survival in HCC patients. Knockdown of *EIF3H* expression in HCC cells promoted apoptosis, and inhibited cell growth, colony formation, migration, as well as xenograft growth. TGF-βand MAPK pathways are potentially targeted by EIF3H.

**Methods:**

*EIF3H* mRNA expression was measured in HCC tissue samples and paired non-tumor samples (N=60) and results were validated in another dataset of 215 HCC patients. Then *EIF3H* expression and clinical outcomes were correlated. Malignant phenotypes were studied after *EIF3H* expression was knocked down with siRNA in HCC cell lines. EIF3H targeted pathways were identified by microarray analysis.

**Conclusion:**

EIF3H is frequently upregulated and is an independent prognostic marker for HCC patients and EIF3H inhibition mitigates the malignant phenotype. Our data provide novel insight into the function of EIF3H in HCC progression, and suggest that EIF3H may be a potentially valuable biomarker for HCC.

## INTRODUCTION

Hepatocellular carcinoma (HCC) is the fifth most common cancer worldwide and the third most common cause of cancer mortality, causing approximately 600,000 deaths annually [1, 2]. HCC is usually diagnosed at an advanced stage when patients are not eligible for curative treatments. Also, the efficacy of systemic chemotherapy is often limited due to drug resistance. Molecular mechanisms underlying these processes are not well understood although numerous genes involved in tumorigenesis and tumor metastasis have been identified. Thus, we have too little molecular data to shape treatment decisions [3].

Dysregulation of protein synthesis has been implicated in oncogenesis via mechanisms whereby “weak” mRNA encoding proteins involved in cell proliferation are translated with activation of the protein synthesis apparatus [4]. mRNAs encoding oncogenic proteins are weak competitors for the translational apparatus relative to other mRNAs, so their translation is less efficient with modest inhibition of protein synthesis, but they are greatly stimulated when overall protein synthesis is activated. Thus, activation of protein synthesis overproduces these oncogenic proteins, and rapid cell growth ensues, whereas down-regulation impairs oncogenic protein syntheses and enables tight control of proliferation. To modify protein synthesis, changing translational apparatus elements or activity, particularly the initiation factors, could be an efficient strategy [5] because the initiation phase is the rate-limiting step for most mRNAs [6]. EIF3 is the largest of the initiation factors, comprising 13 non-identical protein subunits, with a mass approximately 50% less than the 40S ribosomal subunit. Individual over-expression of 6 different EIF3 subunits occurs in various tumors: elevated 3H has been confirmed in 18% of breast and 30% of prostate cancers [7], 26% of hepatocellular carcinomas [8], and in non-small cell lung cancers [9], and colorectal cancers [10, 11]. Elevated EIF3H causes malignant transformation by directly stimulating oncogenic protein synthesis such as cyclin D1, ornithine decarboxylase and fibroblast growth factor 2(FGF-2). Decreasing EIF3H by siRNA knockdown in breast and prostate cancer cell lines slows cell proliferation and reduces anchorage-independent growth in soft agar. These observations indicate that EIF3H is needed to establish and maintain the cancerous state.

In this study, we quantified EIF3H mRNA and protein in HCC tumor tissue, para-cancerous normal tissue, and various HCC cell lines. Using siRNA methods, we noted that depletion of EIF3H in HCC cancer cell lines altered cell malignant phenotypes and reduced *in vivo* HCC tumor growth. These data underscore a role for EIF3H in the etiology and maintenance of HCC.

## RESULTS

### EIF3H expression in HCC

**Figure 1:**
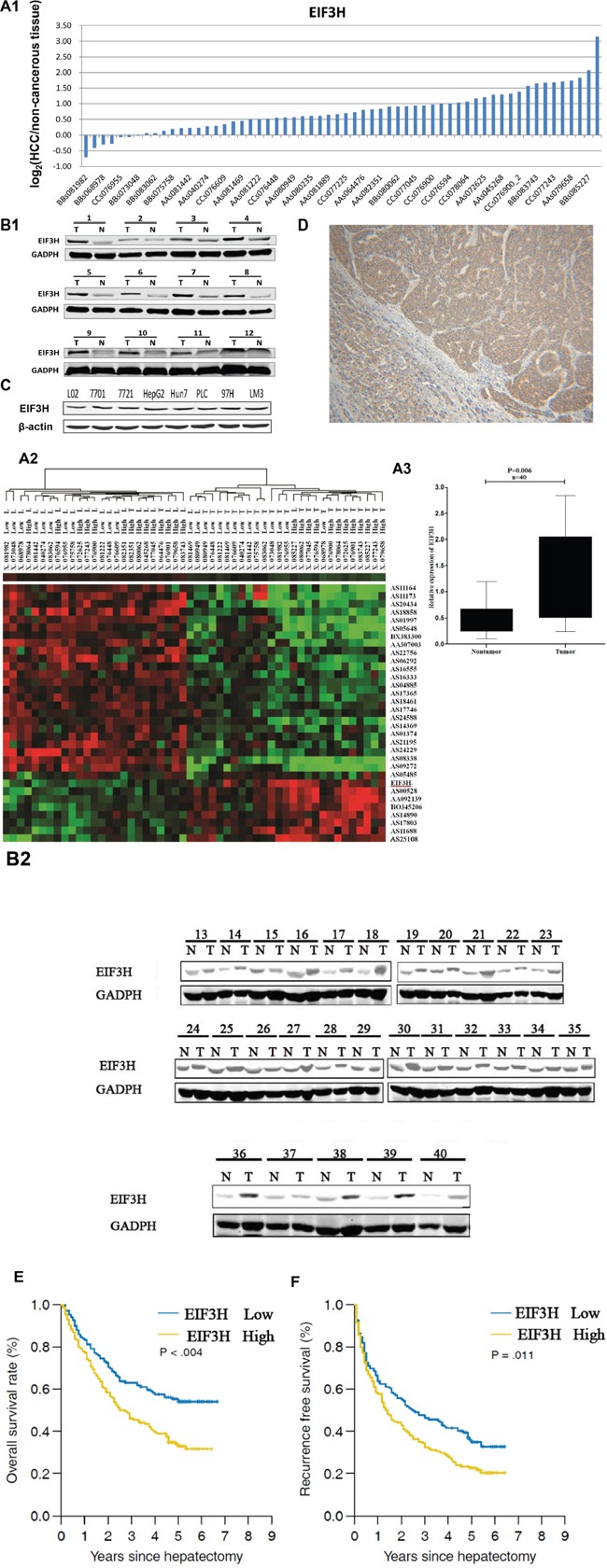
EIF3H was overexpressed and correlated with tumor recurrence and OS of HCC patients **A1.** Quantitative RT-PCR analysis of *EIF3H* in 60 HCC tissues and paired non-tumor tissues, expressed as Log2 (2-ΔΔCt). **A2.** Systemic variations in the expression of EIF3H between 60 HCC samples and the paired non-tumor samples using a microarray analysis. **A3.** The EIF3H is significantly up-regulated in 40 human HCC tissues compared with the corresponding non-cancerous tissues. The statistical differences were analyzed using the Paired t-test. The horizontal lines in the box plots represent the median, the boxes represent the interquartile range, and the whiskers represent the 2.5th and 97.5th percentiles. **B1.** and **B2.**) Western blot shows EIF3H expression was greater in 40 pairs of predominately tumor tissue(T) compared to paired non-tumor tissues(N). **C.** Western blot indicates upregulated EIF3H expression in all tested HCC cell lines. **D.** Representative immunohistology confirms upregulated EIF3H expression in tumors but not in para-cancerous normal tissues. **E.** and **F.** Kaplan-Meier analysis of RFS or OS based on *EIF3H* expression in 215 patients with HCC. Median *EIF3H* was the threshold. HCC patients were *EIF3H* high (greater than median) or low expression groups.

With hierarchical clustering analysis for 60 pairs of HCC tumor and normal tissue samples, we evaluated the role of EIF3H in HCC progression. Data show that *EIF3H* was differentially expressed between HCC and non-tumor samples (Fold change=3.654, P=0.002). Figure 1A1 and 1A2 depict these data. To further validate microarray results, we analyzed EIF3H expression using qRT-PCR and Western blot in 40 pairs of predominately HBV-related HCC and the corresponding peri-tumoral tissues (Supplementary Table 5, cohort 1 and Figure 1B1 and 1B2), and found that EIF3H was significantly up-regulated (P=0.006) in HCC (Figure 1A3). Greater EIF3H protein was observed in cancer patients (Table 1). In addition, upregulated EIF3H protein expression was found in all tested HCC cancer cell lines (Figure 1C). Immunohistochemical analysis agreed with blot data, indicating that EIF3H protein was upregulated in tumor tissues compared to non-tumor tissue (Figure 1D).

**Table 1:** Clinical characteristics of 215 HCC patients according to *EIF3H* expression

Variables	EIF3H subgroups		*p* value
	Low	High	
All cases	107	108	
Age: >53/<53	46/61	38/70	0.241
Gender: male/female	91/16	81/27	0.066
HBs antigen: +/−	95/12	97/11	0.807
Liver cirrhosis: +/−	67/40	73/35	0.444
Serum albumin (g/L): >40/≤40	72/35	84/24	0.085
Serum bilirubin (μmol/L): >17/≤17	25/82	32/76	0.298
HBe antigen: +/−	30/77	39/69	0.205
Tumor size (cm): >5/≤5	35/72	44/64	0.222
Number of tumor: Solitary/Multiple	83/24	85/23	0.841
Edmondson Grade: I+II/III	35/72	39/69	0.601
Micro-vascular Invasion: +/−	22/85	45/63	0.001
Macro-vascular Invasion: +/−	8/99	12/96	0.359
Micro metastases: +/−	43/64	40/68	0.635
Encapsulation: complete/−	52/55	60/48	0.701
TNM stage: I/II/III	78/16/13	57/29/22	0.009
BCLC stage: A/B/C	81/18/8	77/19/13	0.625
ALT (U/L): >40/≤40	43/64	50/58	0.366
AFP (μg/L): >20/ ≤20	73/34	80/28	0.344

### EIF3H is correlated with tumor recurrence and overall HCC patient survival

The expression of EIF3H is upregulated in HCC tissues compared with paired non-tumor tissues from the same donor (Figure 1A-1D), thus linking upregulation of EIF3H to pathological hepatic changes. We measured *EIF3H* expression in tumor tissues from 215 HCC patients with qRT-PCR and median expression was the threshold. Low EIF3H expression in 107 patients was classified as values below the 50^th^ percentile (average ΔCt: 7.012, range: 6.105-8.626, compared with β-actin). High *EIF3H* expression in 108 patients was classified as values above the 50^th^ percentile (average ΔCt = 4.328, range: 2.238-5.627, compared with β-actin). Although *EIF3H* expression was not correlated with gender, age, tumor size or number or serum albumin, bilirubin, HBe or HBs antigen, ALT or AFT, the presence of encapsulation, metastasis, or macro-vascular invasion, or Edmondson grade and BCLC stage, high *EIF3H* expression was more frequently observed in HCC patients with microvascular invasion (*p* =0.001; Table 1) and advanced tumor-node-metastasis T stage (*p* =0.009; Table 1).

A univariate analysis revealed that the gender, tumor size, Edmondson grade, micro-vascular invasion, macro-vascular invasion, pathological satellite, encapsulation, TNM stage, BCLC stage, AFP and *EIF3H* expression were significantly correlated with RFS or OS (Supplementary Table 2). These 11 clinicopathological characteristics were assessed and a multivariate Cox proportional hazards model indicated that high *EIF3H* expression, AFP exceeding 20 μg/L, tumors larger than 5 cm, and the presence of pathological satellite cells were independent risk factors for RFS (Supplementary Table 3). Only high *EIF3H* expression and tumors larger than 5 cm were significant independent factors affecting OS of HCC patients after hepatectomy (Supplementary Table 4).

Patients with early stage HCC, based on BCLC staging have been shown to benefit from radical therapies including hepatectomy. Therefore, we performed Kaplan–Meier analysis of RFS or OS based on *EIF3H* expression in 158 HCC stage A patients. Interestingly, greater *EIF3H* expression was consistently indicative of a shorter OS (Figure 1E, *p* =0.061) and RFS (Figure 1F, *p* =0.011). Thus, EIF3H expression is an independent risk factor for HCC patient OS and RFS.

### Downregulation of *EIF3H* expression with EIF3H-siRNA in HCC cells

**Figure 2:**
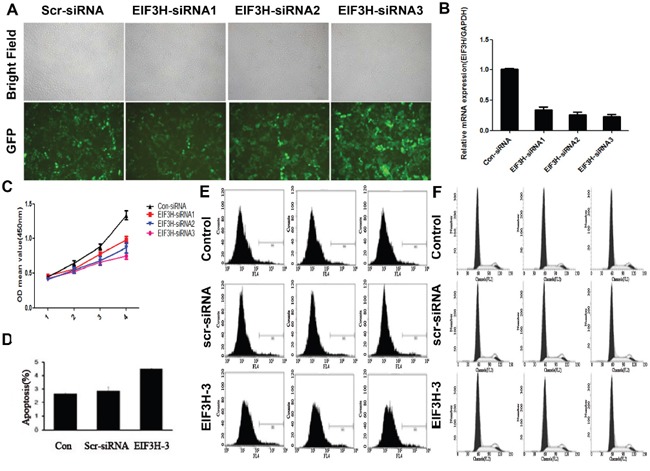
*EIF3H* gene knockdown in HCC cells **A.** Knockdown efficiency of lenti-*EIF3H*-siRNA. HCC cells were treated as described and **B.** *EIF3H* mRNA in EIF3H-siRNA infected and control was analyzed with qRT-PCR. **C.** *EIF3H* gene knockdown inhibited tumor cell proliferation. **D.** and **E.** *EIF3H* knockdown promotes HCC apoptosis. **F.** knockdown of *EIF3H* did not change HCC cell cycle.

**Figure 3:**
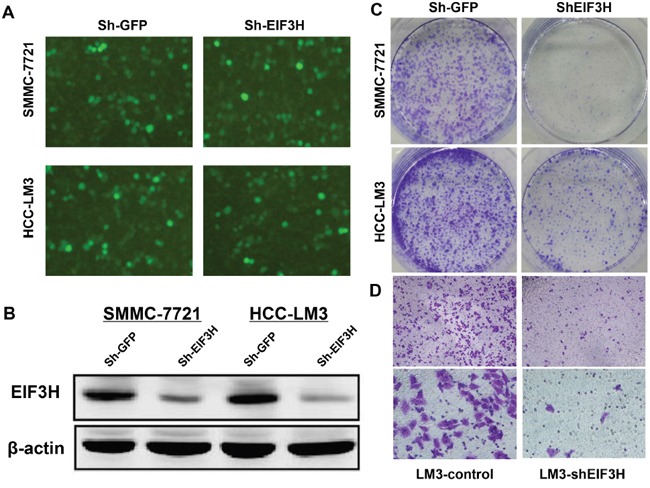
Lentivirus-mediated knockdown of *EIF3H* inhibited colony formation and cell migration **A.** Knockdown efficiency of lenti-*EIF3H*-shRNA in HCC SMMC-7221 and LM3 cells. **B.** EIF3H expression in SMMC-7221 and LM3 cells was reduced after infection with lenti-EIF3H-shRNA according to Western blot. **C.** lenti-*EIF3H*-shRNA infected SMMC-7221 and LM3 formed significantly fewer colonies compared with lenti-sh-*GFP* infected cells. **D.** Transwell assay confirmed significantly less cell migration in lenti-*EIF3H*-shRNA infected SMMC-7221 and LM3 cells.

Lentiviral infection efficiency of HCC cells was measured by microscopic examination of GFP expression (MOI 10). Figure 2A indicates that more than 90% of cells were infected. *EIF3H* mRNA measured with real time RT-PCR were reduced more than 60% in cells infected with *EIF3H*-siRNA1, *EIF3H*-siRNA2, or *EIF3H*-siRNA3 (Figure 2B). Western blot performed 3 days after infection confirmed significant decreases in EIF3H protein expression in *EIF3H*-siRNA infected cells, compared to control scramble siRNA-infected cells (Figure 3B).

### EIF3H knockdown of malignant phenotypes in HCC cells

*EIF3H* knockdown inhibited cell growth, migration, colony formation, and promoted apoptosis (Figure 2C), and this was confirmed with GFP-expressing infected cells that were counted daily for 5 days. All EIF3H-RNAi inhibited cell proliferation in a time-dependent manner. After 5 days of infection, cells were reduced more than 50% compared with control scrambled siRNA-infected cells. *EIF3H*-RNAi-3 was the most potent (92.8%), followed by *EIF3H*-RNAi-2 (65.6%) and *EIF3H*-RNAi-1 (52.1%).

*EIF3H* knockdown modified cell cycle progression and apoptosis in HCC cells and provided mechanistic data. Figure 2D and 2E indicate that *EIF3H* knockdown resulted in increased apoptosis. Apoptotic cells were 5.22%, 6.07%, and 9.05% in control, scramble-siRNA, and *EIF3H*-siRNA infected cells (p=0.001), respectively. No significant differences in cell cycle progression, including G_0_/G_1_, S, and G_2_/M phases, were noted between cells infected with *EIF3H*-siRNA, scramble-siRNA, or control cells (Figure 2F).

We assessed lentiviral infection efficiency in Sh-*EIF3H* in HCC SMMC-7721 and HCC-LM3 cells with microscopic examination of GFP expression (MOI 10). Figure 3A shows that more than 90% of cells were infected. Western blot, performed 3 days after infection, confirmed a significant decrease in EIF3H protein expression in *EIF3H*-siRNA infected cells, compared to control scramble siRNA infected cells (Figure 3B). After 14 days of infection, cell colonies in Sh-*EIF3H*-infected SMMC-7721 and HCC-LM3 cells were reduced by 53.4% and 62.5% compared with sh-*GFP* infected cells, respectively (Figure 3C). Furthermore, after shRNA-silencing of *EIF3H* migration and invasion assays revealed that SMMC-7721 and HCC-LM3 migration decreased by 91.0% (Figure 3D). Thus, EIF3H is vital for HCC cells to migrate and may have a role in HCC metastasis.

### EIF3H knockdown modified tumor growth in a xenograft mouse model

**Figure 4:**
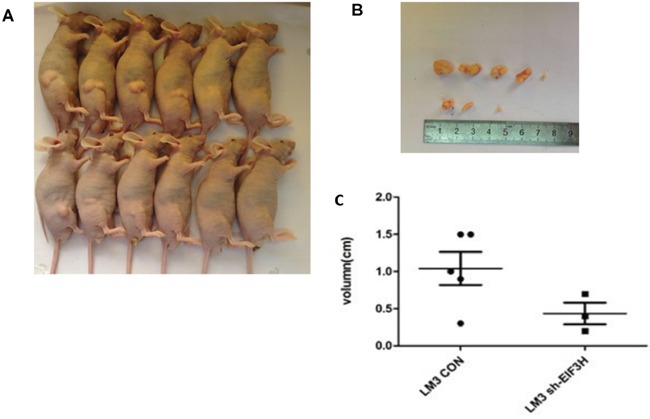
Lentivirus-mediated knockdown of *EIF3H* inhibited *in vivo* LM3 tumor growth **A.** Representative images indicate reduced tumor growth in mice inoculated with lenti-*EIF3H*-shRNA infected LM3 cells. **B.** and **C.** At week 4 after inoculation, xenograft tumors were photographed and measured. The length of the tumors from the two groups was plotted (p<0.05).

To study the effect of lentivirus-mediated *EIF3H*-siRNA on tumor growth in a xenograft mouse model, 1 week after tumor cell inoculation, we noted tumor growth and 4 weeks after inoculation, five of the six tumor formation in mice of control group was detected and measured, while three of the six were detected and measured in EIF3h knock-down group of mice as shown in Figure 4, xenograft tumor size was as follows (mean ± SD): 1.07 ± 0.49 in control shRNA infected LM3 cell group, and 0.49± 0.19 in the shRNA infected LM3 cell group (Figure 4A, 4B and 4C). A two tailed t-test confirmed inhibition of tumor growth after *EIF3H* knockdown that was significant compared with controls (p=0.005).

### Microarray analysis identified genes and pathways potentially targeted by EIF3H

**Figure 5:**
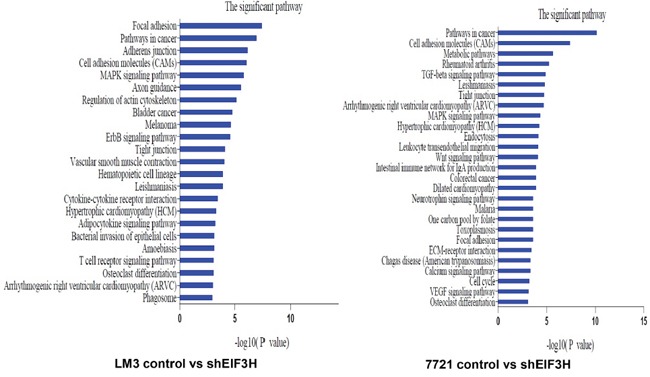
Kyoto Encyclopedia of Genes and Genomes (KEGG) pathway analysis of EIF3H knock-down or control cells lines *P* values < 0.05 and false discovery rates < 0.05 were used as thresholds to select significant KEGG pathways. The horizontal axis denotes the LgP (log (*P* value)) of significant pathways, with LgP indicating the logarithm of each *P*-value to the base 10. The significance of specific pathways in EIF3H knock-down cell lines compared to control is denoted by -LgP.

To identify EIF3H targeted genes and pathways in HCC, total RNA was extracted from scrambled control siRNA and *EIF3H*-siRNA infected SMMC-7221 and LM3 cells, and was hybridized with a Human Genome U133 Plus 2.0 array containing 47,000 transcripts, representing 38,500 Entrez genes. Among them, mRNAs were confirmed to be differentially expressed genes and potential EIF3H targets. With Kyoto Encyclopedia of Genes and Genomes (KEGG) analysis, 22 pathways were identified to be up-regulated or down-regulated in *EIF3H* knockdown LM3 cells. The top five significant pathways were: focal adhesion, adherens junction, cell adhesion molecules (CAMs), MAPK signally pathways, and axon guidance. In SMMC-7221 cells, 21 pathways were identified to be up- or down-regulated by *EIF3H* silencing. The top five pathways were: cell adhesion molecules (CAMs), metabolic pathways, rheumatoid arthritis, TGF-beta signaling pathway, and Leishmaniosis (Figure 5).

### *EIF3I* expression with EIF3H-siRNA in HCC cells

**Figure 6:**
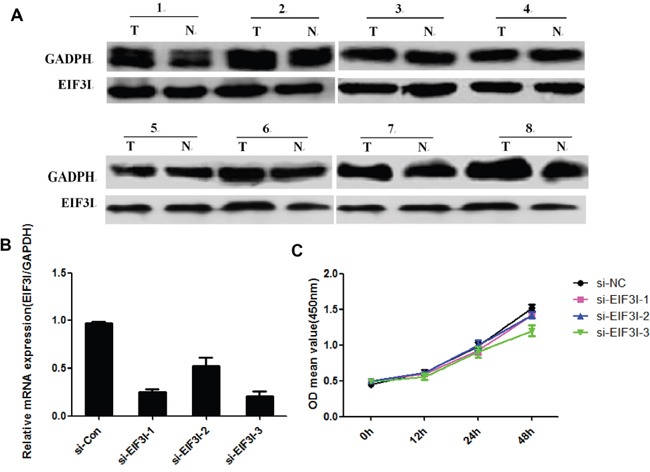
**A.** EIF3I expression by western blotting in HCC tissues. **B.** and **C.** Cancer cells proliferation related to EIF3I knock down.

We investigated EIF3I, an EIF3 member of module components expression by western blotting in HCC tissues. There was no difference of EIF3I expression was detected between tumor and para-tumor (Figure 6A), suggesting that EIF3H was separated from other EIF3 subunits in HCC. We also performed siRNA target for EIF3I to investigate its role in regulation of cancer cell proliferation. As shown in Figure 6B and 6C, EIF3I knock down has modest effect on cancer cells proliferation. Therefore, in contrast to other EIF3 subunits, over-expression of EIF3H on cells proliferation in HCC was specific.

## DISCUSSION

Although advances have been made in HCC diagnosis and treatment, patient prognosis is poor [15–18]. Sorafenib, the first and only approved targeted therapy for HCC was developed to disrupt the Ras-Raf-MEK1/2-ERK1/2 signaling pathway by specifically targeting Raf-1 kinase and its chemotherapeutic efficacy was correlated with inhibition of MEK and ERK phosphorylation, and other receptor tyrosine kinases, such as VEGFR-2 and 3 [19–21]. Sorafenib offers molecularly targeted therapy, but efficacy decreases over time due to drug resistance *via* escape/compensatory mechanisms. So, to improve patient prognosis, better drugs are needed and this will be best achieved by better understanding molecular mechanisms of hepatocarcinogenesis.

Over the past decade, the contribution of eukaryotic translation initiation factor 3 (EIF3), to malignant transformation and progression has been defined. A role for EIF3 in cancer derives in large part from the observation that many tumors appear to over-express or under-express a specific subunit of EIF3. For example, high levels of EIF3a have been reported in breast, cervix, esophagus, lung and stomach cancers. Similar individual over-expression in specific cancers (such as bladder, colon, breast and prostate cancers) is reported for the 3b, 3c, 3h, 3i and 3m subunits. In addition, ectopic over-expression of some of these subunits in immortal cell lines can cause malignant transformation. Increased EIF3 stimulates protein synthesis and preferentially activates oncogenic RNAs. A recent siRNA screening study identified eight driver genes, including *EIF3H*, on chromosome 8q23, that regulate the proliferation/survival of clonogenic breast cancer cells. Overexpression of *EIF3H* does not lead to elevated EIF3, but protein synthesis is enhanced, as is translation of oncogenic mRNAs. Indeed, knockdown of expression of these genes decreased cell viability via both cell cycle arrest and induced apoptosis, and this inhibited colony formation under anchorage-independent conditions in soft agar [22].

However, an EIF3 subunit promotes cancer are probably weak, as rigorous controls may be lacking or alternative interpretations are not ruled out, and how the EIF3H subunit stimulates protein synthesis and promotes malignant transformation of immortal cells is unclear. Moreover, little research has been reported to explain how the human *EIF3H* gene in participates in HCC development and treatment. Thus, to address this gap in our understanding, we recognized the limitations of previous experiments used in studying EIF3 in cancer, then applied this to examine the evidence linking EIF3H subunit to HCC. Most importantly, we first measured differential expression of EIF3H in HCC tumor samples and non-tumorous normal tissue samples and we associated elevated EIF3H expression and clinical outcomes in early stage HCC patients. Data from 60 paired HCC tumor samples and para-cancerous normal tissue samples confirmed that *EIF3H* mRNA was significantly differentially expressed (Fold change=3.654, P=0.002) in HCC tumor tissue. EIF3H overexpression was also observed in HCC cell lines. We confirmed this in another dataset of 215 paired HCC and normal tissue samples, and noted that EIF3H expression is upregulated in HCC tissue compared with the paired non-tumor tissues. In patients with BCLC Stage A HCC, greater expression of *EIF3H* was associated with shorter OS and more cancer recurrence compared with patients with less expression of EIF3H.

Using lentiviral-mediated siRNA to inhibit *EIF3H* expression in SMMC-7721 and LM3 HCC cell lines, we noted that more than 90% of cells remained (MOI 10) on day 5 after infection. *EIF3H*-siRNA infection effectively reduced expression of EIF3H in both cell lines as confirmed by qRT-PCR and Western blot. Carcinogenesis typically unbalances cell proliferation and cell death and our data confirmsignificant inhibition of HCC cell growth due to *EIF3H* knockdown. This inhibition appeared to be due to apoptotic induction, but not cell cycle arrest in both SMMC-7221 and LM3 HCC cells. Furthermore, LM3 cells infected with siRNA-*EIF3H*, and then inoculated into nude mice grew more slowly than control LM3 cells. Thus, functional knockdown of EIF3H expression inhibits HCC growth and metastasis *in vitro* and *in vivo*.

The association between *EIF3H* expression and poor prognosis may be due to “strong and weak mRNAs”. Translation of strong mRNAs (e.g. those encoding housekeeping proteins such as β-actin) will quickly reach a maximum with low free EIF3H whereas weak mRNAs are poorly translated. With more free, activated EIF3H, the translation of “weak mRNAs” relating to malignancy is disproportionately enhanced, which contributes to malignant activity [23, 24]. To understand how EIF3H mitigates HCC malignant phenotypes, microarray analysis was used to identify genes and pathways potentially targeted by EIF3H. In *EIF3H*-siRNA infected SMMC-7221 and LM3 cells, KEGG enrichment analysis identified 22 and 21 pathways affected by *EIF3H* silencing, respectively. Among these pathways, MAPK and Wnt-β-catenin offered the greatest support foran important role for EIF3H in the regulation of cell signaling networks. The compounds that target pathways in hepatocarcinogenesis, such as IGF and cixutumumab, as well as the Wnt/*β*-catenin pathways are under early-stage evaluation in HCC.

The selections of treatment modalities of HCC include liver surgery, transarterial chemoembolization (TACE), ablation, radiation and immunotherapy. Surgery or transplantation remain the mainstays of curative therapy for early disease. Ablative strategies can also cure small tumors. TACE and external beam radiation therapy can control locally advanced disease no longer amenable to cure, the use of doxorubicin-loaded bead-based TACE tends to be offered to patients with more extensive HCC or higher risk of systemic toxicity. Exploring biomarkers early on is a big step forward. We will follow with interest immunotherapeutic strategies, such as nivolumab, an anti-PD-1 antibody [25], and GC33, a novel recombinant humanized monoclonal antibody, but a strategy for a phase III trial has not yet been optimized.

Wagner and colleagues found that a, b, c, g, and i subunits constitute some sort of a primordial EIF3 that gradually evolved by adding more subunits with extra functions to cope with increasing complexity of life [26]. Thus, it would be essential to investigate if EIF3H overexpression might go along and is driven by an additional overexpression of an EIF3 member of module I. In our study, we investigated EIF3I, an EIF3 member of module components expression by western blotting in HCC tissues. There was no difference of EIF3I expression was detected between tumor and para-tumor (Figure 6A), suggesting that EIF3H was separated from other EIF3 subunits in HCC. We also performed siRNA target for EIF3I to investigate its role in regulation of cancer cell proliferation. As shown in Figure 6B and 6C, EIF3I knock down has modest effect on cancer cells proliferation. Therefore, in contrast to other EIF3 subunits, over-expression of EIF3H on cells proliferation in HCC was specific.

*EIF3H* overexpression increases tumor cell proliferation, growth, and survival. Overexpression of *EIF3H* is documented in prostate, breast, and liver cancer [7, 27], and is associated with advanced cancer stage and poor prognosis in prostate cancer [27]. Reduction of EIF3H has previously been shown to reduce cell proliferation and anchorage-independent growth in soft agar [27]. We confirm that manipulating *EIF3H* expression has a similar effect in HCC and our data provide compelling evidence that high EIF3H directly stimulates protein synthesis, resulting in the establishment and maintenance of the malignant HCC phenotype. Strategies for inhibiting the expression of these genes or protein function encoded by these genes may have value for treating liver cancers as a therapeutic target or by serving as a biomarker through the identification of amplifications of corresponding genomic regions.

## MATERIALS AND METHODS

### Cell lines and tumor samples

All cell lines including HCC LM3, SMMC7721, LO2, 97H, Hep3B, HepG2, and Huh7 were purchased from American Type Culture Collection (Manassas, VA). All cell lines were maintained by serial passaging in the appropriate media containing 10% fetal bovine serum at 37°C in a humidified atmosphere of 5% CO_2_ and 95% air. We collected 60 HCC tissues and paired non-cancerous tissue samples for microarray analysis. Another 215 HCC tissues were used for validation of microarray data by quantitative real-time PCR analysis (qRT-PCR) and survival analysis. All samples were obtained from patients undergoing hepatectomy at the Eastern Hepatobiliary Surgery Hospital between March 1 and April 30, 2008. Human tissue use was approved by the local ethics committee and patients gave written informed consent. Only patients with clear pathological HCC diagnosis and who had not received neoadjuvant chemotherapy or radiotherapy were enrolled. Patients who died from non-hepatic disease or accidents, and those without complete clinical data and laboratory tests were excluded. Immediately after resection, tumor tissue and para-cancerous normal tissue at least 2 cm away from the tumor border from the same patient were collected, snap-frozen and stored in liquid nitrogen until use. HCC patient clinical characteristics are listed in Table 1. Tumor differentiation was classified according to the Edmondson grading system (I, well-differentiated; II, moderately differentiated; III, poorly differentiated; IV, undifferentiated). Tumor staging was defined according to the 6^th^ edition of the Tumor-Node-Metastasis (TNM) classification system published by the International Union against Cancer and the BCLC staging system [12]. Micro-metastases were defined as tumors adjacent to main tumor border that could be identified microscopically.

### Follow-up

Patient follow-up was performed every 2-3 months during the first year after surgery and 3-6 months thereafter until October 31, 2014. The median follow-up duration was 46 months (range 6–80 months). All follow-up examinations were performed by two physicians blinded to study data. All patients were monitored by abdomen ultrasonography, chest X-ray, and measurement of serum AFP every month during the first year after surgery and every 3-6 months thereafter. A computed tomography (CT) scan or magnetic resonance imaging (MRI) of the abdomen was performed every 6 months or immediately after a recurrence was suspected. Diagnostic criteria for recurrences were equal to that for preoperative diagnosis. Once recurrent tumors were confirmed, treatment was implemented based on tumor diameter, number, location, and vessel-invasion and hepatic function data. Recurrence-free survival (RFS) was calculated from the date of tumor resection until tumor recurrence or the last observation. Overall survival (OS) was defined as the length of time between surgery and death or the last follow-up examination.

### Tissue microarray construction and immunohistology

The tissue microarray (TMA) was constructed as described previously [13]. In brief, all samples were reviewed histologically with hematoxylin and eosin staining. Representative areas were premarked on paraffin blocks, away from necrotic and hemorrhagic tissue. Two 1.0-mm cores were extracted from each tumor, paired with non-tumor tissue, and mounted on a new recipient block with a semi-automated array device (TMArrayer, Pathology Devices, Westminster, MD).

Immunohistochemistry was performed on the TMA with a two-step Envision technique [13]. Briefly, after heating the sections in 10 mmol/L citrate buffer for antigen retrieval, sections were incubated sequentially with primary antibody and EnVision reagent (HRP-M) at room temperature. Then, sections were developed in a diaminobenzidine solution under a microscope and counter-stained with hematoxylin. Immunohistochemical stains were assessed by two separate pathologists blinded to patient characteristics. Expression was evaluated based on staining intensity of positive cells and the percent of positive cells in the section (negative = none; +, weak signal; ++, intermediate signal intensity; +++, strong signal intensity).

### Quantitative real-time PCR

Total RNA from cell lines and tissue samples was assessed with TRIzol reagent according to the manufacturer's instructions (Thermo Scientific, Wilmington, DE). Quality, integrity and quantification of total RNA were measured spectrophotometrically with NanoDrop (Thermo Scientific) and gel electrophoresis. cDNA synthesis was performed with the SuperScript III cDNA synthesis kit (Life Technologies, Grand Island, NY). Specific primers for human GAPDH and EIF3h were used. qRT-PCR was conducted using an Applied Biosystems ABI Prism 7500 detection system (Life Technologies). Then, a 20 μL reaction mixture was prepared in duplicate, containing 1 μL of complementary DNA, 10 μL of SYBR Green PCR Master Mix and 1 μL (10 μM) of each primer. Thermal cycler conditions included an initial activation step at 95°C for 10 minutes, followed by a 3-step PCR program of 95°C for 15 seconds, 60°C for 20 seconds, and 72°C for 20 seconds for 40 cycles. The 2^-ΔΔct^ method was used for relative quantification of gene expression, and data were normalized to β-actin expression.

### Immunoblotting

Proteins from SDS gels were transferred to polyvinylidene difluoride membranes and immunoblotted with antibodies indicated in the figures, followed by incubation with the corresponding secondary antibodies (Sigma-Aldrich, St. Louis, MO). Signals were measured by incubating the blots with 5-bromo-4-chloro-3-indolyl-phosphate/nitroblue tetrazolium (Sigma-Aldrich). Blots were scanned with a photoscanner and quantified with Scion Image software. Antibodies used included monoclonal anti-EIF3h (Santa Cruz Biotechnology, Santa Cruz, CA) and anti-actin (Sigma).

### Stable knock-down expression of EIF3H in HCC with lentiviral vectors

Small interfering RNAs (siRNAs) targeting the EIF3H gene were designed by the Shanghai He-Yuan BioTech, Co. Ltd, China. Three optimal sequences of siRNA against human EIF3H (5′-GTGCTTTTGGGTCTGGTTGT-3′) were then cloned into the plasmid GV112. Lentiviral preparations were produced by Shanghai GeneChem, Co. Ltd, China. Resulting shRNA human EIF3H sequences were confirmed by PCR and sequencing analysis. The primers and siRNA used are presented in Supplementary Table 1. To produce recombinant lentiviruses, HEK293T cells were co-transfected with respective recombinant expression lentivectors in combination with enveloped and packaged plasmids using Lipofectamine™ 2000 transfection reagent (Life Technologies). The viral supernatant was harvested 48 h after transfection, and viral titer wasquantified. Viral supernatant was added into target HCC cells (MOI 10) with ENi. S and 5 μg/mL polybrene to obtain stably-transfected HCC cells with knocked down *EIF3H*. Two controls were established by transfecting a negative control siRNA with a scrambled sequence with no known homology to human, mouse, or rat sequences or by transfecting with green fluorescence protein (GFP).

### Characterization of malignant phenotypes in HCC cell lines

To investigate malignant phenotypic changes after downregulating EIF3H in HCC cell lines we preformed experiments described below in triplicate or three times independently.

First, cell growth was measured with a cell counting kit-8 (CCK8, Sigma-Aldrich) with water soluble tetrazolium salt WST-8 ([2- (2-methoxy-4-nitrophenyl)-3- (4-nitrophenyl)-5- (2,4-disulfophenyl)-2H-tetrazolium, monosodium salt]). Briefly, HCC cells infected with lentivirus-mediated *EIF3H*-siRNA or control siRNA were seeded in 96-well plates (2×10^3^ cells/well) and cultured at 37°C in a 5% CO_2_ humidified incubator. Infected GFP-expressing cells were imaged and counted in parallel. Growth curves of infected cells were plotted.

Next, to analyze cell growth, colony formation analysis was performed with HCC cells infected with lentivirus-mediated EIF3H-siRNA or control siRNA which were seeded in 6-well plates (1,000 cells/well). Cells were cultured for 14 days at 37°C in a 5% CO_2_ humidified incubator. Culture medium was changed every 3 days. Cell colonies were imaged and counted manually.

Then, the cell cycle distribution was assessed with DNA staining with propidium iodide (PI) and flow cytometry. In brief, HCC cells seeded in 6 cm culture dishes were infected with lentivirus-mediated EIF3H-siRNA or control siRNA. After infection, cells were trypsinized, washed with PBS, and fixed with 70% ethanol for at least 1 h at 4°C. After two washing steps in cold PBS, cells were resuspended in 0.5 mL of PBS containing 100 μg/mL RNase A and 50 μg/mL PI, and incubated for 30 min in the dark at 4°C. Cells in different cell cycle phases were measured with flow cytometry and Modfit software.

An Annexin V apoptosis assay was used to measure apoptosis. Briefly, HCC cells seeded in 6-well plates were infected with lentivirus-mediated EIF3H-siRNA or control siRNA. Then, attached cells were trypsinized, washed with PBS and centrifuged. Cells were washed with 1× binding buffer, centrifuged, and resuspended in 1 mL 1x binding buffer. To 100 μL of cell suspension, 5 μL Annexin-V-APC was added, followed by gentle vortexing, and 10 min incubation at room temperature in the dark. Data acquisition and analysis were performed using flow cytometry and dedicated software.

A cell migration assay using a Millicell 24-well culture insert plate (Millipore, city, state) and polycarbonate membranes (pore size 8 μm) were used in Transwell assays as described {Hu, 2004 #124}. First, the insert plates were equilibrated with 0.6 mL of medium for 1 h at 37°C in 5% CO_2_. Then 1×10^5^ cells suspended in 0.4 mL serum-free medium were added into the upper chambers, whereas complete medium containing 10% FBS was added into the lower chamber of each well. After 24 h incubation, insert plates were rinsed with PBS and the upper surface of the membranes were scraped using a cotton swab to remove cells. Cells on the underside of the membranes (containing migrated cells) were fixed with paraformaldehyde (4%) and stained with crystal violet (0.01%). Pictures were obtained at 10x magnifications. Cells in six different fields were counted.

### *In vivo* xenograft tumor models

All animal experiments were approved by the Institutional Animal Care and Use Committee of the Second Military Medical University, Shanghai, China. Four- to five-week-old female nude mice were purchased from the Shanghai Laboratory Animal Center of the Chinese Academy of Science. To establish xenografts, mice were anaesthetized with 2% isoflurane and 1×10^7^ SMMC-LV-sh-EIF3h or SMMC-LV-sh-NC cells were injected (sc) at the left or right shoulder, respectively. Tumor volumes were measured every three days (volume =1 /2 × length × width × depth).

### Microarray-based gene expression profiling and data analysis

Gene expression was measured in EIF3H-siRNA and control siRNA infected HCC cells and compared using a Human Genome U133 Plus 2.0 Microarrays Kit (Agilent Technologies, Santa Clara, CA). Microarrays were performed following the manufacturer's protocol. RNA quantity and purity were determined by optical density measurements and RNA integrity by using the 2100 Bioanalyser (Agilent Technologies, CA). Only high quality RNA was further processed. Briefly, total RNA was extracted, purified and characterized and then hybridized to the Human Genome U133 Plus 2.0 Array. After hybridization, the array was washed and processed using an Agilent DNA microarray scanner. Feature Extraction Software (FES) was used to read and process the microarray image files. GeneSpring GX v12.1 was used to confirm feature intensities and ratios. Quantile normalization was conducted and only gene changes detected from at least one of six samples were subsequently analyzed. Differentially expressed genes were identified through the random variance model. A *P* value was calculated using the paired t-test. The threshold set for up- and down-regulated genes was a fold change ≥ 2.0 and a *P* value < 0.05. The Kyoto Encyclopedia of Genes and Genomes (KEGG) analysis for differentially expressed genes was performed using NIH gene annotation software. Heat maps were presented using cluster 3.0. Genes with statistically significant differential expression according to fold-change values were summarized and represented with volcano plots. Confirmed DEGs were subjected to GO functional enrichment analysis [14].

### Statistical analysis

The threshold value of EIF3H expression was determined by using ROC (receiver operating characteristic) curve analysis. RFS and OS were defined as the interval between the date of diagnosis to the first HCC recurrence (local or distant) or to the subject's death from HCC, respectively. Survival curves were calculated using the Kaplan-Meier method, and differences were assessed with a log-rank test. The Cox proportional hazards model was used to measure independent factors based on variables selected by univariate analysis. For statistical comparisons, ANOVA, the chi-square test, Fisher's exact test, or a two-tailed Student's t-test were performed when appropriate. All statistical analyses were performed with SPSS version 16.0 software. *P* values less than 0.05 was considered to be statistically significant.

## SUPPLEMENTARY MATERIALS TABLES



## Abbreviations

EIF3H: eukaryotic translation initiation factor 3 subunit H
HCC: hepatocellular carcinoma
qRT-PCR: quantitative real-time polymerase chain reaction
TNM: Tumor-Node-Metastasis
BCLC: Barcelona clinic liver cancer
RFS: recurrence-free survival
OS: overall survival
ALT: alanine aminotransferase
AFP: alpha-fetoprotein
HR: hazards ratio
CI: confidence interval.
