# Supplementary material for: Elevated expression of eukaryotic translation initiation factor 3H is associated with proliferation, invasion and tumorigenicity in human hepatocellular carcinoma
Source: Oncotarget. 2016 Jun 22;7(31):49888–901. doi: 10.18632/oncotarget.10222 (PMC5226555; doi:10.18632/oncotarget.10222)
Supplement: Supplementary file 1 [file oncotarget-07-49888-s001.pdf]

Elevated expression of eukaryotic translation initiation factor 3H is associated with proliferation, invasion and tumorigenicity in human hepatocellular carcinoma

SUPPLEMENTARY TABLES

Supplementary Table S1: Sequences of primers and siRNA used

Genes		Sequence
EIF3H	Sense primer	GTGCTTTTGGGTCTGGTTGT
	Anti-sense primer	AGCCCACGTGAAGATGATCA
GAPDH	Sense primer	GCACCGTCAAGGCTGAGAAC
	Anti-sense primer	TGGTGAAGACGCCAGTGGA
EIF3H-siRNAs	SiRNA-1	GCAAGTGCAGATAGATGGC
	SiRNA-2	GTGCAGATAGATGGCCTTG
	SiRNA-3	GAAGAAGGACAAGGAACTG

Supplementary Table S2: Univariate analysis of clinical outcomes of 215 HCC patients

Variable	RFS (%)*	P value	OS (%)*	P value
Age: >53/<53	51.8/49.2	0.721	77.8/69.9	0.268
Gender: male/female	46.4/68.4	0.021	70.6/82.7	0.221
HBs antigen: +/-	51.5/45.6	0.325	76.9/66.6	0.109
Liver cirrhosis: +/-	52.1/49.6	0.732	72.5/77.8	0.589
Serum albumin (g/L): >40/≤40	51.5/48.6	0.627	71.0/75.2	0.616
Serum bilirubin (μmol/L): >17/≤17	53.7/45.7	0.063	70.1/75.0	0.320
HBe antigen: +/-	34.0/66.0	0.000	59.2/89.1	0.000
Tumor size (cm): >5/≤5	53.5/43.6	0.236	76.0/62.1	0.065
Number of tumor: Solitary/Multiple	61.2/44.5	0.015	82.5/69.5	0.024
Edmondson Grade: I+II/III	25.4/59.9	0.000	49.7/83.5	0.000
Micro-vascular Invasion: +/-	16.3/54.8	0.000	49.6/76.0	0.024
Satellite	30.3/61.5	0.000	62.9/81.7	0.002
Encapsulation: complete/-	41.5/59.7	0.005	65.5/83.9	0.001
TNM stage: I/II/III	64.5/52.3/17.4	0.000	85.8/57.0/46.3	0.000
BCLC stage: A/B/C	57.6/35.1/16.4	0.000	78.5/61.0/50.1	0.001
ALT (U/L): >40/≤40	48.5/52.6	0.539	71.0/74.9	0.644
AFP (μg/L): >20/ ≤20	42.1/68.7	0.001	70.2/83.9	0.023
EIF3H	61.5/40.2	0.000	79.9/66.0	0.005

RFS: Recurrence-Free Survival; OS: Recurrence-Free Survival; *the time follow-up ended is used to calculate the Recurrence-Free Survival and Overall survival; p value by Long-rank test less than 0.05 was considered statistical significant.

Supplementary Table S3: Multivariate analysis for factors related to HCC RFS

Variable	Univariate			Multivariate		
	HR	95% CI	P value	HR	95% CI	P value
Age(year) >53/ ≤53	1.002	0.991-1.012	0.737			
Gender: male/femal	1.214	1.048-2.635	0.018	1.199	0.808-1.781	0.397
HBsAg: positive/negative	1.339	0.941-1.905	0.138			
HBeAg: positive/negative	1.196	0.906-1.580	0.186			
Liver cirrhosis: +/-	1.263	0.956-1.670	0.098			
Antiviral therapy	1.290	0.932-1.784	0.122			
TBL(μmol/L): >17 / ≤17	0.989	0.961-1.019	0.476			
ALB (g/dL):>40 / ≤40	0.982	0.942-1.023	0.386			
ALT (U/L): >40 / ≤40	1.001	0.997-1.006	0.477			
TNM stage: I/ II/ III	1.612	1.296-3.148	0.006	1.012	0.986-1.023	0.446
Number of tumor: Solitary / Multiple	1.056	0.934-1.136	0.423			
BCLC stage: A/B/C	1.764	1.372-3.721	0.004	0.946	0.649-1.381	0.780
Edmondson-Steiner grade: I + II/III	1.573	1.282-3.521	0.015	1.088	0.827-1.430	0.548
Tumour encapsulation:No/ Complete	1.832	1.346-2.567	0.031	1.122	0.843-1.493	0.435
Micro-vascularinvasion(+/-)	1.593	1.392-2.764	0.012	1.290	0.932-1.784	0.122
Macro-vascularinvasion(+/-)	1.843	1.251-2.682	0.037	1.339	0.941-1.905	0.138
EIF3H	1.411	1.064-1.871	0.017	1.979	1.297-2.996	<0.001
AFP(≤20ug/L *vs* >20ug)	1.634	1.120-2.385	0.011	1.841	1.062-3.167	0.0260
Tumor size (≤5 cm *vs*>5 cm)	1.957	1.444-2.654	<0.001	2.539	1.520-4.256	<0.001
Satellite (presence *vs* absence)	2.2820	1.7111-3.0435	<0.001	2.358	1.460-3.702	0.0020

HCC: Hepatocellular carcinoma; HR: Hazard ratio; CI: Confidence interval; TBL: Total bilirubin; ALB: Albumin; ALT: Alanine aminotransferase; PT: Prothrombin time; PLT: Blood platelet; AFP: Alpha-fetoprotein.

Supplementary Table S4: Multivariate analysis for factors related with HCC OS

Variable	Univariate			Multivariate		
	HR	95%CI	P value	HR	95%CI	P value
Age(year) >53/ ≤53	1.023	0.891-1.412	0.268			
Gender: male/femal	1.241	1.108-1.842	0.021	1.024	0.732-1.204	0.371
HBsAg: positive/negative	1.134	0.654-1.487	0.109			
HBeAg: positive/negative	1.785	0.896-4.382	0.081			
Liver cirrhosis: +/-	1.084	0.695-1.423	0.589			
Antiviral therapy	1.082	0.341-1.396	0.267			
TBL(μmol/L): >17/ ≤17	0.765	0.201-1.138	0.320			
ALB (g/dL):>40/ ≤40	0.934	0.672-1.104	0.784			
ALT (U/L): >40/ ≤40	1.003	0.984-1.014	0.841			
TNM stage: I/II/III	2.434	1.783-3.627	0.001	1.145	0.863-1.616	0.061
Number of tumor: Solitary/ Multiple	1.172	0.734-2.354	0.074			
BCLC stage: A/B/C	2.821	1.452-3.783	0.001	1.106	0.721-1.854	0.104
Edmondson-Steiner grade: I + II / III	2.153	1.637-4.526	0.001	1.004	0.632-1.657	0.568
Tumour encapsulation:No/ Complete	2.145	1.738-3.728	0.001	1.013	0.832-1.751	0.168
Micro-vascularinvasion(+/-)	1.379	1.168-2.872	0.024	1.109	0.768-1.892	0.117
Macro-vascularinvasion(+/-)	1.572	1.265-2.783	0.036	1.125	0.547-2.691	0.276
EIF3H	1.527	1.237-2.351	0.005	1.927	1.775-2.582	0.002
AFP(≤20ug/L*vs* >20ug/L)	1.568	1.254-2.249	0.023	1.035	0.836-1.762	0.126
Tumor size (≤5 cm *vs*>5 cm)	1.752	1.267-2.893	0.035	2.134	1.628-3.458	0.001
Satellite (presence *vs* absence)	1.882	1.543-2.762	0.012	1.258	0.837-1.453	0.076

HCC: Hepatocellular carcinoma; HR: Hazard ratio; CI: Confidence interval; TBL: Total bilirubin; ALB: Albumin; ALT: Alanine aminotransferase; PT: Prothrombin time; PLT: Blood platelet; AFP: Alpha-fetoprotein.

Supplementary Table S5: Clinicopathological characteristic of HCC patients included

Variable	Cohort 1♦	Cohort 2▲	Cohort 3●
All cases	40	215	65
Age(year): >53/<53	16/24	85/130	20/45
Gender: male/female	31/9	180/35	49/16
HBs antigen: +/-	37/3	195/20	55/10
Liver cirrhosis: +/-	29/11	141/74	44/21
Serum albumin (g/L): >40/≤40	31/9	157/58	40/25
Serum bilirubin (μmol/L): >17/≤17	10/30	57/158	15/50
HBe antigen: +/-	16/24	70/145	16:39
Tumor size (cm): >5/≤5	14/26	75/140	27/38
Number of tumor: Solitary/ Multiple	32/8	171/44	55/10
Edmondson Grade: I+II/III	7/33	68/147	25/40
Micro-vascular Invasion: +/-	11/29	65/150	20/45
Macro-vascular Invasion: +/-	4/36	19/196	8/57
Pathological Satellite: +/-	15/25	88/127	28/37
Encapsulation: complete/-	25/15	120/95	36/29
ALT (U/L): >40/≤40	18/22	98/117	35/30
AFP (μg/L): >20/ ≤20	35/5	159/56	40/25

Patients of group ▲were independent from patients of group ♦, and patients of group ● were selected from group ▲ with well-preserved serum.
